# Challenging the dogma to “always operate” acute hip fractures: a proof-of-concept pilot study for nonoperative management of undisplaced femoral neck fractures

**DOI:** 10.1186/s13037-022-00324-x

**Published:** 2022-04-21

**Authors:** Juan Manuel Vinas-Rios, Jan-Henning Wölm, Richard Martin Sellei, Andreas Ladenburger

**Affiliations:** 1 Department of Orthopaedics and Traumatology Sana Klinikum Offenbach, Offenbach am Main, Germany; 2 Department of Orthopaedics and Traumatology, Sanaklinik Offenbach am Main, Starkenburgring 66, 63069 Offenbach am Main, Germany

**Keywords:** Undisplaced femoral neck fracture, Conservative treatment, Fracture impaction

## Abstract

**Background:**

The notion that all acute hip fractures are a surgical entity requiring either surgical fracture fixation or hip replacement represents a historic dogma, particularly within the orthopaedic community of the United States. The present study from a European regional trauma center was designed to challenge the notion that stable and undisplaced femoral neck fractures represent an absolute indication for surgical management.

**Methods:**

The purpose of this study was to investigate the hypothesis that stable and undisplaced femoral neck fractures of the Garden types 1 and 2 can be safely managed nonoperatively. A retrospective observational cohort study was carried out at a regional orthopaedic trauma center in Germany from January 1, 2016 to June 30, 2021. The inclusion criteria specified patients older than 18 years suffering a < 24 h, traumatic, femoral neck fracture Garden types 1 and 2. Exclusion criteria included Garden types 3 and 4 femoral neck fractures, pregnancy, active infection or previous surgery, tumor-associated fractures, medical history of femoral neck necrosis, vascular injury associated with femoral neck fractures, nerve injury associated to a femoral neck fracture and ≥ 24 h femoral neck fracture. The primary intention of this research was to identify deterioration of fracture retention with an ensuing unplanned trip to the operating room in femoral neck fractures Garden types 1 and 2. Secondary were included unplanned readmissions and complications such as surgical site infection.

**Results:**

A total of 41 undisplaced femoral neck fractures (Garden types 1 and 2) were included in this study; *n* = 20 were in the resulting admission operatively treated (group 1) and *n* = 21 were treated conservatively. The mean age in group 1 was 76 years; women (70%). In group 2 it was 81 years with a female dominance (71.4%). Admission status: Garden types 1 and 2, group 1 *n* = 13/7 and group 2 *n* = 15/6. Subsequent femoral neck fracture displacement (Y/N) (in case of operation, before operation) group 1 *n* = 14/6 and group 2 *n* = 6/15.

**Conclusion:**

According with our results, patients sustaining Garden type 1 femoral neck fractures, depending on age and comorbidities, should be treated conservatively with weight bearing and under physiotherapeutic instructions. In case of femoral neck fractures Garden type 2, a surgical treatment should be performed in order to avoid femoral neck fractures to slip after weight bearing by lacking of fracture impaction.

## Background

Currently it is still controversial, that all acute hip fractures are a surgical entity requiring either surgical fracture fixation or hip replacement, illustrating a historic dogma, particularly within the orthopaedic community of the United States [1–4]. The present study from a European regional trauma center was designed to challenge the belief that stable and undisplaced femoral neck fractures type Garden types 1 and 2 represent an absolute indication for surgical management [1–3]. In 2014, the American Association of Orthopaedic Surgeons (AAOS) released a Clinical Practice Guideline (CPG) on Management of Hip Fractures in the Elderly as follows [2]:

- Regional analgesia with fascia iliaca blocks can improve preoperative pain, timing of surgery is important; improved outcomes are seen if surgery can be performed within 24 to 48 h [3, 4].
- Moderate evidence supports operative fixation for patients with stable (undisplaced) femoral neck fractures [5–9].
- Multimodal pain control in the elderly can minimize delirium, improve patient satisfaction, decrease complications, and improve early mobility [6, 9].
- Bipolar heads do not appear to provide any advantage, so using unipolar heads is a good value-based recommendation unless costs are comparable [10, 11].

Despite the above mentioned considerations, it remains unclear whether conservative treatment should be used to treat the common undisplaced femoral neck fractures (Garden classification, Garden types 1 and 2) [3–8]. Here, we review German patients after conservative and surgical treatment of undisplaced femoral neck fractures.

The treatment options are conservative (bed rest with or without traction) and surgical (internal fixation) [3, 4]. Surgical treatment has been reported to be optimal [1–4]. However, any surgery is associated with inherent risks such as infection and thromboembolic event [1–4, 7, 8]. Nevertheless, patients undergoing conservative treatment showed good outcomes in selected studies [7, 8].

## Methods

The purpose of this study was to investigate the hypothesis that stable and undisplaced femoral neck fractures of the Garden types 1 and 2 can be safely managed nonoperatively. A retrospective observational cohort study was carried out at a regional orthopaedic trauma center in Germany from January 1, 2016 to June 30, 2021. The inclusion criteria specified patients older than 18 years suffering a < 24 h, traumatic, femoral neck fracture Garden types 1 and 2. In the course of the above inclusion criteria was decided whether the initially conservatively treated patients would require surgery or continue to be conservatively treated based on the re-evaluation of the clinical and radiological findings.

Patients with the following characteristics were excluded:

- Femoral neck fractures Garden types 3 and 4
- Pregnancy.
- Active infection or previous surgery.
- Tumor-associated fractures
- Medical history of femoral neck necrosis.
- Vascular injury associated to femoral neck fractures
- Nerve injury associated to femoral neck fracture
- ≥24 h femoral neck fracture

The primary objective of this research was failure of fracture retention with a consequently unintentional trip to the operating room in femoral neck fractures Garden types 1 and 2. Secondly included were unplanned readmissions, surgical site infection, mobilization and pain on discharge. Dementia and previous mobility were recorded. The limb weight-bearing and mobility under conservative therapy were documented. In particular, it was evaluated whether a discharge from the clinic was possible and whether resumption took place due to the fracture. The pain level became quantitative with the visual analog scale (VAS) detected. Additionally, the period since the traumatic event was recorded as well as which type of surgical procedure was performed.

## Results

In total, 41 initially conservative treated undisplaced femoral neck fractures (Garden types 1 and 2) were included in this study; *n* = 20 were in the following admission operatively treated (group 1) and *n* = 21 were conservatively managed. The mean age in group 1 was 76 years; women (70%). In group 2 was 81 years with female dominance (71.4%). Admission status: days until operation in group 1 *n* = 17.7 ± 9.8 days; Garden type 1, group 1 *n* = 13 and group 2 *n* = 15 and Garden type 2, group 1 *n* = 7 and group 2 *n* = 6. Dementia was found in 3 patients in group 1 and 5 patients in group 2. The admission status and clinical evaluation is as follows: Operative treatment rejection by admission group 1 *n* = 7, group 2 *n* = 1 and subsequent femoral neck fracture displacement (Y/N) group 1 *n* = 14/6 and group 2 *n* = 6/15 (see Table 1).

**Table 1:** Demographics, classification, clinical evaluation by admission and discharge from patients undergoing surgery by femoral neck fracture and those treated conservatively

	Group1 Unplanned surgery, after initial nonoperative management due to secondary fracture displacement (***n*** = 20)	Group2 Nonoperative (***n*** = 21)	
Gender (M/F)	6/14	6/15	
Age (years) (Median, range)	76 ± 8.4	80.9 ± 9.2	
**Admission Status**			
Days until operation	17.7 ± 9.8	N/A	
Garden Type 1	13	15	
Garden Type 2	7	6	
Dementia (Y/N)	3/17	5/16	
Operative treatment rejection by admission	7	1	
Follow-up femoral neck fracture displacement (Y/N) (in case of operation, before operation)	14/6	6/15	
**Weight Bearing**			
Partial/touchdown weight bearing	17	18	
Non-weight bearing	10	0	
Full weight bearing	7	13	
Pain by discharge (≥ 5 VAS^a^)	5	7	

^a^Visual analog scale

On the other hand, patient’s weight bearing were: mobilization with partial weight bearing group 1 *n* = 17, group 2 *n* = 18; non-weight bearing group 1 *n* = 10, group 2 *n* = 0, full weight bearing group 1 *n* = 7, group 2 *n* = 13 and pain on discharge ≥5 visual analog scale (VAS) group 1 *n* = 5, group 2 *n* = 7 (Table 1).

The type of surgery in patients with operative treatment in the subsequent admission was as follows: Hemiarthroplasty in *n* = 12, total arthroplasty *n* = 3, Dynamic Hip Screw (DHS) *n* = 4 and gamma nail in a highly progredient displacement of the fracture with per trochanteric component *n* = 1.

## Discussion

This study of more than 40 patients within a more than 4 year time frame provided a rare treatment modality of femoral neck fractures namely conservative treatment. Actually, although the AAOS’s recommendation of conservative treatment for femoral neck fractures Garden types 1 and 2 in selected patients, the great majority will be surgically treated in one way or another [1–4]. In our point of view, the highlight of this study lies mainly in the number of patients initially treated conservatively. Therefore, such a casuistic with this amount of patients is rare nowadays. The main point of this study lies in avoiding bearing weight of the affected extremity after a femoral neck fracture Garden types 1 and 2 [10–14]. Those patients without an impacted femoral neck fracture are prone to have a slip of the fracture by further weight bearing [15, 16]. Furthermore, we recommend that patients with an impacted femoral neck fracture, specially Garden types 1 to be treated conservatively from the beginning regardless of the health status and age of the patient [17–20]. However, biomechanical studies have confirmed that fracture fixation and immobilization affect the pattern of skeletogenic stem cell differentiation into osteoblasts; mechanical fixation will obviously influence neovascularisation [11, 12, 21] that could be simulated in the impaction of the fracture in those treated conservatively with further compression and fixation as seen in Garden type 1 femoral neck fracture. Thus, impaction promotes bone union [21]. In some studies, the union rates reached were as high as 90% [19, 22–24]. Further advantages achieved by avoiding surgery in patients with impacted fractures include lack of bleeding, wound-healing problems and infection of the surgical site [25, 26].

In comparison with our results, most studies revealed a significant risk of displacement during nonoperative treatment. The risk of displacement varied from 14.1 to 55.7% [11, 24, 27]. Verheyen et al. [27] explored the rate of secondary displacement in 105 patients. Forty-eight patients (46%) were at risk of such displacement; however, the patient group had a high mean age with non-impacted fractures. Therefore, secondary displacement was more common in patients aged >70 years with non-impacted fractures, in agreement with the data of Raaymakers [11], who reported secondary instability in 41% of patients >70 years of age.

Hence the main key for treatment selection is a precise diagnosis prior to choosing a treatment option [15, 16, 21, 28]. Radiography has certain limitations when used to distinguish femoral neck fracture types, which can result in misdiagnosis. Furthermore, a patient may in fact have a non-impacted femoral neck fracture but be diagnosed as having an impacted one [4–6, 15, 16, 21, 28]. In addition, diagnoses using the Garden classification are very inconsistent, although widely performed. We recommend a CT-Scan for patients sustaining femoral neck fractures Garden types 1 and 2 for more understanding of the morphology of the femoral neck fracture with possible impaction or not, together with further treatment options [4–6, 15, 16, 21, 29].

In our study patients who underwent an unplanned surgery after initial nonoperative management due to secondary fracture displacement was on average 17 days. Furthermore, it has been demonstrated that patients not being operated on within the next 24 h after suffering a displaced femoral neck fracture are potentially at risk of developing severe complications such as pneumonia and deep venous thrombosis with subsequent death [30]. However, this allegation is particularly crucial in patients with comorbidities, advanced age (>70 years old) [12] and femoral neck fractures Garden types 3 and 4 where an operative procedure is regarded in the majority of the cases as the unique type of treatment. Furthermore, these claims could be interpreted with caution in patients with femoral fracture particularly in Garden type 1 with subsequent fracture impaction, where a conservative treatment has been described in patients <70 years old or without comorbidities. Our group of patients, in particular those with a femoral fracture Garden type 1 with a valgus-impacted stable hip fracture were allowed to fully weight bear. Even more, in our study, patients <70 years old with no comorbidities, were treated conservatively after sustaining a Garden type 1 femoral neck fracture with fracture impaction, whilst the majority of patients further treated surgically, were Garden type 2 femoral fractures. Thus, according with our results, Garden Type 2 femoral neck fractures were prone to slip after weight bearing, being a characteristic of crucial importance for unplanned surgery, after initial non-operative management due to secondary fracture displacement in not valgus-impacted stable hip fracture [12–14].

This work has obvious limitations: Firstly it’s retrospective study nature. It could negatively affect the accuracy of our findings. A future strictly designed and adequately powered RCT is essential. Secondly: It recorded Garden types 1 and 2 femoral neck fractures; their prognoses would differ, according to fracture impaction, greatly from each other. A future study could compare treatment outcomes among patients with these two types of femoral neck fractures and their grade of impaction.

However, it is worth comparing the two treatment protocols in view of cost-benefits. In nonoperative treatment hospital costs are usually higher, as a result of prolonged bed rest, longer rehabilitation, frequent checkups, and an increased incidence of local and general complications such as pneumonia and deep venous thrombosis in older patients (70 years) with comorbidities [12]. The costs of operative management (anaesthesia, implants, etc.) are returned by a shorter hospitalization, and the avoidance of complications of longer recumbency [25, 26].

From our results, the following asseverations can be summarized:

1. Early mobilization alongside adequate multimodal analgesic therapy could play a crucial role in further secondary fracture displacement with consequent unplanned surgery, after initial nonoperative management in the first days after trauma in Garden type 2 femoral neck fractures.
2. CT-Scans should be performed in patients sustaining femoral neck fractures Garden types 1 and 2 for more understanding of the morphology of the femoral neck fracture in order to detect possible impaction with further treatment options.
3. Patients with non-impacted femoral neck fractures particularly Garden type 2 should be treated surgically depending on age and preoperative status.

## Conclusion

According with our results, patients sustaining Garden type 1 femoral neck fractures, depending on age and comorbidities, should be treated conservatively with weight bearing and under physiotherapeutic instructions. In case of femoral neck fractures Garden type 2, a surgical treatment should be performed in order to avoid femoral neck fractures to slip after weight bearing by lacking of fracture impaction.

## Abbreviations

AAOS: American Association of Orthopaedic Surgeons
CI: Confidence interval
CPG: Clinical Practice Guideline
CT: Computer Tomography
DHS: Dynamic Hip Screw
MRI: Magnetic Resonance Imaging
OR: Likelihoods ratio
RCT: Randomized control trial
RR: Relative risk
SD: Standard Deviation
VAS: Visual analog scale
